# Supplementary material for: Prevalence and correlates of contraceptive use among adolescent mothers: Results from a cross-sectional survey in Ouagadougou, Burkina Faso, and Blantyre, Malawi
Source: PLoS One. 2025 Jun 18;20(6):e0319136. doi: 10.1371/journal.pone.0319136 (PMC12176169; doi:10.1371/journal.pone.0319136)
Supplement: Attachment — Submitted filename: PLOS ONE Comments- 3-25-25.docx [file pone.0319136.s001.docx]

**Abstract**

l68- replace would with may or could

l72- remove various

Results: results for 19yrs & < 16 given- readers may wonder re ages 16-18.

**Intro:**

L68-70- doesn’t flow easily for the reader; would reword to clarify what you’re trying to convey

L68- change would to may

L81- you refer to ages 15-24- your upper age for adolescence is confusing & seems out of place with rest of paper

L86- provider bias – clarify r/t to subject of this paper-e.g. reluctance to provide contraceptives to adolescents????

LL98-1-101- This sentence should be rephrased, e.g. We sought to conduct a study that would contribute to the broader global efforts….. or moved to beginning of discussion. The end of the intro should begin with a sentence that includes the purpose of this study, e.g. We designed a cross sectional descriptive study to …….

**Materials & Methods**: should start with a brief summary of the previous study as described in lines 106- 109 …We used data obtained from a larger mixed methods study……. and then continue with …. You may need to provide a brief explanation about using a two-stage stratified sampling approach to survey the adolescent girls in BF & Malawi

LL 119-120 is the purpose of your study and also belongs at the end of the introduction; just before you state that you designed a cross sectional descriptive study to….

Line 121- clarify what you mean by nuanced in this context

Line 129- term enumeration areas is not clear (at least not to me)

L 135- household listing exercise unclear; next line explains a sampling from that you used- how does this differ from the household listing exercise. Would simplify the language for this methodology

LL 139-141- Would use was sufficient but not say based on the study’s research question-; would summarize as expressed at the end of the introduction (you never clearly state a research question; you’re investigating the prevalence and correlates of contraceptive use among parenting adolescent girls in urban & rural settings….. . As above would put the research goal at the end of the intro. You could also Sart with the sentence beginning on line 139, e.g. Based on the formula for calculating the sample size for an observation study………, we estimated that a sample size of 500 adolescent mothers is sufficient….

L 147- Is there a ref for the pretested questionnaire?

L153- Spell out APHRC (first use) African Population and Health Research Center

LL Outcome Variables- Did you provides samples or pictures of the various methods you asked about?

LL173-187- Which variables were considered Community level covariates?

**RESULTS:**

L201- Where did most of the participants live (in both countries most didn’t live with parents)

Table 4 shows some ever used differences for 18 yos – consider a mention in results

L255- Use Women of Islamic religion (not Islamic religion) had a sisgnificantly lower odds of contraceptive use…….

**Discussion:**

L 14- Reword first sentence- In this study we examined …..The results showed

L 17- Higher proportion (higher than who/where???)

Would consider adding to the Discussion some suggestions for further research including . the role of parents (especially mothers) in adolescent opinions and use of contraceptives as well as the role of male partners.
