# Supplementary material for: Prevalence and correlates of contraceptive use among adolescent mothers: Results from a cross-sectional survey in Ouagadougou, Burkina Faso, and Blantyre, Malawi
Source: PLoS One. 2025 Jun 18;20(6):e0319136. doi: 10.1371/journal.pone.0319136 (PMC12176169; doi:10.1371/journal.pone.0319136)
Supplement: Attachment — Submitted filename: Reviewer-Editor comments.docx [file pone.0319136.s005.docx]

# Prevalence and correlates of contraceptive use among parenting adolescents: results from a cross-sectional survey in Ouagadougou, Burkina Faso, and Blantyre, Malawi

## Response to reviewers

### Editor’s comments

#### Abstract

| *Reference (lines)* | *Comment* |
| --- | --- |
| *Results “Parenting girls” – line 35 - 36* | *Not clear what this means: pre-teenagers and adolescents?* |
| *Response* | |
| ***Thank you for bringing this up, we omitted the word -adolescent- it was meant to read as “parenting adolescent girls”. This has been changed to “adolescent mothers” throughout the paper.*** | |
| *Reference (lines)* | *Comment* |
| *Results describing odds of contraceptive use* | *...compared to...? (Unclear)* |
| *Response* | |
| ***The comparisons in this section are focused on marital status (single vs married) and age (19 years vs younger)*** | |
| *Reference (lines)* | *Comment* |
| *Results describing infrequent use of contraceptives* | *"64.9%" is high! Please check or explain.* |
| *Response* | |
| ***This section describes the reasons for the non-use of contraceptives among non-users. Furthermore, our results show that in Malawi, the surveyed adolescent mothers reported infrequent sex as the main reason for not using contraceptives, indicating they do not need it due to not being at risk of falling pregnant. The main difference between Malawi and Burkina Faso is that adolescent mothers are far more likely to be in stable relationships in Burkina Faso than in Malawi.*** | |
| *Reference (lines)* | *Comment* |
| *Conclusion* | *"Adolescent mothers" or all adolescents?* |
| *Response* | |
| ***These recommended intervention avenues are targeted at adolescent mothers but have implications for their male counterparts*** | |

#### Main body

| *Reference (lines)* | *Comment* |
| --- | --- |
| *Title* | *Look at the suggestions done to the Abstract, above.* |
| *Response* | |
| ***With thanks, the comments from the abstract were helpful, the title has changed slightly to reflect this. The paper is around adolescent mothers (or parenting adolescent girls).*** | |
| *Reference (lines)* | *Comment* |
| *Introduction (81)* | *"South Saharan Africa (SSA)" [Use the full expression before the acronym, the first time over]* |
| *Response* | |
| ***We appreciate that you highlighted this, a correction has been effected.*** | |
| *Reference (lines)* | *Comment* |
| *Study participant and sampling (91)* | *Explain this term for the reader...* |
| *Response* | |
| ***The lived experience of pregnant and parenting adolescents survey refers to a research study that was part of a larger research and advocacy project. We understand that stating it in this manner could have the potential to confuse the reader; to that effect, we have clarified the term to make it accessible and void of ambiguity.*** | |

| *Reference (lines)* | *Comment* |
| --- | --- |
| *Study participant and setting (130)* | *Any explanation for the selection of the EAs* |
| *Response* | |
| ***We selected the enumeration areas (EAs) in line with the sampling frame provided by the Malawi statistical office, as well as the Burkina Faso National Institute of Statistics and Demography. In the first stage, rural and urban enumeration areas (EAs) were randomly selected, 71 EAs in Burkina Faso (29 rural EAs and 42 urban EAs) and 66 EAs in Malawi (26 rural EAs and 40 urban EAs).*** | |

| *Reference (lines)* | | *Comment* |
| --- | --- | --- |
| *Data collection (153)* | | *Put the full expression first time, before the acronym...* |
| *Response* | | |
| ***We appreciate that you highlighted this, a correction has been effected*** | | |
| *Reference (lines)* | *Comment* | |
| *Outcome variable (170)* | *What about the "rhythm method", one of the most commonly used traditional method?* | |
| *Response* | | |
| ***The rhythm method was indeed part of the available options within the questionnaire tool. For the purposes of brevity and conciseness, this was not mentioned. We have, however, included it at this point.*** | | |
| *Reference (lines)* | *Comment* | |
| *Independent variable (185)* | *Did no one had "superior" (post-secondary)?* | |
| *Response* | | |
| ***As at the time of the study, and our inclusion criteria, quite a few of the adolescent mothers were out of school and very few completed secondary school. A negligible number either attempted or completed post-secondary. Specifically, 3 adolescents in Malawi Faso attained technical with no secondary, secretary training, university and 4 (post-secondary) in Burkina Faso. They were ultimately grouped into the secondary/higher category*** | | |

| *Reference (lines)* | | *Comment* |
| --- | --- | --- |
| *Table 1 - education* | | *This adds to more than 100%. Please check.* |
| *Response* | | |
| ***Thank you for picking up on this, this was a typographical error during the compilation of the table*** | | |
| *Reference (lines)* | *Comment* | |
| *Results (230)* | *What about the rhythm methods which appear in the table? (Important the big difference between ever use and current use...* | |
| *Response* | | |
| ***The big difference is due to method switch based interaction with the clinics during ANC and method discontinuation.*** | | |
| *Reference (lines)* | *Comment* | |
| *Results (261)* | *Important to interpret this finding: religious observance could influence differently in different countries...is it associated with differences in cultures, other factors?...* | |
| *Response* | | |
| ***This is indeed an important point for interpretation. With appreciation, we agree that an interpretation of this paradoxical finding is needed, and we have gone ahead to do this as part of a corresponding paragraph in the discussion section. See below:***  ***The divergent findings underscore that religion does not operate in a vacuum but interacts with cultural systems, gender norms, and healthcare access. Islamic teachings in Burkina Faso often emphasize traditional family values, including early marriage and pronatalist norms (prioritizing childbearing). Conservative interpretations of Islam may discourage contraceptive use, viewing it as interfering with divine will (Qadar) or as promoting "immoral" sexual autonomy outside marriage. Whereas some Islamic leaders in Malawi have endorsed contraception for spacing births (consistent with the Quranic principle of maternal and child health). Organizations like the Islamic Medical Association of Malawi actively promote family planning as compatible with faith, emphasizing ‘azl (withdrawal), a historically permitted method. In Burkina Faso, Islam’s majority status reinforces conservative interpretations tied to cultural identity. In Malawi, Muslims’ minority status may foster adaptation to majority-Christian norms or health systems. These contrasts highlight the need for context-specific interventions that engage religious leaders, address structural barriers, and leverage local cultural frameworks to improve reproductive health outcomes.*** | | |
| *Reference (lines)* | *Comment* | |
| *Results (p25) line 1* | *", the..." (no need to use a period if the sentence has not been completed)* | |
| *Response* | | |
| ***Thank you for picking up on this, this is another typographical error*** | | |
| *Reference (lines)* | *Comment* | |
| *Discussion (29)* | *Although spousal support is in places not as high as one would expect, which affects the type of contraception being used (e.g., injectables instead of pills).* | |
| *Response* | | |
| ***Thank you for this proposition. It makes a fine addition to the discussion. And has been included as the concluding sentence.*** | | |
| *Reference (lines)* | *Comment* | |
| *Strength and limitations (61 – 70)* | *Yes, it is important to highlight this more because the study seems to indicate a high use of contraception, while in the country as a whole it is more likely that there is even far more nonuse and more need for education, promotion and services than this study reveals...!* | |
| *Response* | | |
| ***We appreciate this point of view and agree that this position be amplified. To that effect, we have highlighted this further in the discussion section.*** | | |

#### Reviewer #1

Abstract

| *Reference (lines)* | *Comment* |
| --- | --- |
| L68/Introduction | replace would with may or could |
| *Response* | |
| ***Thank you for this observation, a correction has been effected.*** | |
| *Reference (lines)* | *Comment* |
| L72 | remove *various*  Results: results for 19yrs & < 16 given- readers may wonder re ages 16-18. |
| *Response* | |
| ***Thank you for this observation. The initial intention was to highlight the age extremes and leave readers to fill in the blanks with the results subsequently provided. In agreement with the reviewer’s stance and we have provided some results for the 16 – 18 group.*** | |

#### Introduction

| *Reference (lines/section)* | *Comment* |
| --- | --- |
| *Ll68-70/introduction* | *doesn’t flow easily for the reader; would re-word to clarify what you’re trying to convey* |
| *Response* | |
| ***We greatly appreciate that you captured this discrepancy. We have rephrased that paragraph to be simpler and easier to follow. We removed redundancy and repetition to allow for better flow and clarity.*** | |
| *Reference (lines/section)* | *Comment* |
| L68 | change would to may |
| *Response* | |
| ***We have changed this and acknowledge that we were speaking with unmeasurable certainty rather than possible probabilities.*** | |
| *Reference (lines/section)* | *Comment* |
| L81- | you refer to ages 15-24- your upper age for adolescence is confusing & seems out of place with the rest of paper |
| *Response* | |
| ***With appreciation to this comment, we have changed the age comparison and provided a different reference.*** | |

| *Reference (lines/section)* | *Comment* |
| --- | --- |
| L86- | provider bias – clarify r/t to subject of this paper-e.g. reluctance to provide contraceptives to adolescents???? |
| *Response* | |
| ***Yes, provider bias describes the reluctance of health care providers to provide/prescribe contraceptives to adolescents. We agree that some level of clarification was required here and have added a phrase to qualify that description.*** | |
| *Reference (lines/section)* | *Comment* |
| LL98-1-101- | This sentence should be rephrased, e.g. We sought to conduct a study that would contribute to the broader global efforts….. or moved to beginning of discussion. The end of the intro should begin with a sentence that includes the purpose of this study, e.g. We designed a cross sectional descriptive study to ……. |
| *Response* | |
| ***Thank you for identifying this issue. This is a very important observation that structures the manuscript, making it clearer and accessible. We have moved the first sentence you referenced to the start of the discussion and concluded the introduction section with the purpose of the study.*** | |

#### Materials and methods

| *Reference (lines/section)* | *Comment* |
| --- | --- |
| 106- 109 | should start with a brief summary of the previous study as described in lines …We used data obtained from a larger mixed methods study……. and then continue with …. You may need to provide a brief explanation about using a two-stage stratified sampling approach to survey the adolescent girls in BF & Malawi |
| *Response* | |
| ***We appreciate that you highlighted this, a correction has been effected.*** | |
| *Reference (lines/section)* | *Comment* |
| LL 119-120 | is the purpose of your study and also belongs at the end of the introduction; just before you state that you designed a cross-sectional descriptive study to…. |
| *Response* | |
| ***As highlighted above, the concluding remark in the introduction now aligns with this comment. Describing the purpose of the study.*** | |
| *Reference (lines/section)* | *Comment* |
| Line 121- | clarify what you mean by nuanced in this context |
| *Response* | |
| ***Nuanced in this context refers to the subtle differences noticeable spanning the two countries where the study was conducted. These include characteristics like Residence, marital status as well as their reactions to contraception as a reflection of their religion.*** | |
| *Reference (lines/section)* | *Comment* |
| Line 129- | term enumeration areas is not clear (at least not to me) |
| *Response* | |
| ***An enumeration area (EA) is a geographic unit used primarily for census and survey purposes. It is the smallest geographic area into which a country is divided for the collection of population and housing data.*** | |

| *Reference (lines/section)* | *Comment* |
| --- | --- |
| L 135 | - household listing exercise unclear; next line explains a sampling from that you used- how does this differ from the household listing exercise. Would simplify the language for this methodology |
| *Response* | |
| ***Thank you for highlighting this important aspect of the research. For the two-stage sampling strategy, the first stage involved random sampling of urban and rural enumeration areas (EAs). In the second stage, we conducted a household listing in the sampled EAs to identify households with eligible pregnant and parenting adolescent girls. We have attempted to simplify the language in this regard to make the process clear for the reader.*** | |
| *Reference (lines/section)* | *Comment* |
| LL 139-141- | Would use was sufficient but not say based on the study’s research question-; would summarize as expressed at the end of the introduction (you never clearly state a research question; you’re investigating the prevalence and correlates of contraceptive use among parenting adolescent girls in urban & rural settings….. . As above would put the research goal at the end of the intro. You could also Sart with the sentence beginning on line 139, e.g. Based on the formula for calculating the sample size for an observation study………, we estimated that a sample size of 500 adolescent mothers is sufficient…. |
| *Response* | |
| ***The approach used to address this concern falls in line with the previous comment on the purpose of the study appearing at the end of the introduction. We indeed agree that for the purposes of this paper the broader project’s research questions were different. As such we have carefully rephrased the sampling process.*** | |
| *Reference (lines/section)* | *Comment* |
| L 147 | - Is there a ref for the pretested questionnaire? |
| *Response* | |
| ***Thank you for this question; We adapted this questionnaire from the DHS additionally, we are happy to share our pre-tested questionnaire as part of the submission for this manuscript. This questionnaire is quite long and therefore we are more likely to provide it on request made directly to the corresponding author.*** | |
| *Reference (lines/section)* | *Comment* |
| L153 | - Spell out APHRC (first use) African Population and Health Research Center |
| *Response* | |
| ***We appreciate that you highlighted this, a correction has been effected*** | |

| *Reference (lines/section)* | *Comment* |
| --- | --- |
| LL Outcome Variables | - Did you provides samples or pictures of the various methods you asked about? |
| *Response* | |
| ***We provided pictures, and each field interviewer was required to ensure that the participants had a working knowledge of the forms of contraception before proceeding with the line of questioning.*** | |
| *Reference (lines/section)* | *Comment* |
| LL173-187- | Which variables were considered Community level covariates? |
| *Response* | |
| ***Only place of resident is considered a community level covariate.*** | |

Results

| *Reference (lines/section)* | *Comment* |
| --- | --- |
| L201- | Where did most of the participants live (in both countries most didn’t live with parents) |
| *Response* | |
| ***Most participants resided with their partners (if married or living like married) and quite a number lived alone particularly those in urban environments. The majority resided within their local communities and villages not too far from extended families. Unless under exigent circumstances e.g. unknown and denied paternity of the child few adolescent girls lived far from their place of birth.In Burkina Faso, approximately four out of five adolescent mothers (82%) reported not living with their parents, but only about 50% reported not living with their parents in Malawi.*** | |
| *Reference (lines/section)* | *Comment* |
| Table 4 | shows some ever used differences for 18 yos – consider a mention in results |
| *Response* | |
| ***Thank you for highlighting this, we have included a mention in the results*** | |
| *Reference (lines/section)* | *Comment* |
| L255 | - Use Women of Islamic religion (not Islamic religion) had a significantly lower odds of contraceptive use……. |
| *Response* | |
| ***We appreciate that you highlighted this, a correction has been effected.*** | |

#### Discussion

| *Reference (lines/section)* | *Comment* |
| --- | --- |
| L 14- | Reword first sentence- In this study we examined …..The results showed |
| *Response* | |
| ***We appreciate that you highlighted this, a correction has been effected.*** | |
| *Reference (lines/section)* | *Comment* |
| L 17 | - Higher proportion (higher than who/where???) |
| *Response* | |
| ***When compared to the national averages in both countries, adolescent mothers in our study have a higher prevalence of CP use.*** | |
| *Reference (lines/section)* | *Comment* |
| *General* | Would consider adding to the Discussion some suggestions for further research including. the role of parents (especially mothers) in adolescent opinions and use of contraceptives as well as the role of male partners. |
| *Response* | |
| ***We have added this suggestion, thank you for this input.*** | |
